# Study on Thermal Decomposition Behaviors of Terpolymers of Carbon Dioxide, Propylene Oxide, and Cyclohexene Oxide

**DOI:** 10.3390/ijms19123723

**Published:** 2018-11-23

**Authors:** Shaoyun Chen, Min Xiao, Luyi Sun, Yuezhong Meng

**Affiliations:** 1 College of Chemical Engineering and Materials Science, Quanzhou Normal University, Quanzhou 362000, China; chshaoy@qztc.edu.cn; 2 The Key Laboratory of Low-carbon Chemistry & Energy Conservation of Guangdong Province/State Key Laboratory of Optoelectronic Materials and Technologies, Sun Yat-Sen University, Guangzhou 510275, China; stsxm@mail.sysu.edu.cn; 3 Department of Chemical & Biomolecular Engineering and Polymer Program, Institute of Materials Science, University of Connecticut, Storrs, CT 06269, USA; luyi.sun@uconn.edu

**Keywords:** polycarbonate, thermal decomposition kinetics, TG/FTIR, Py-GC/MS

## Abstract

The terpolymerization of carbon dioxide (CO_2_), propylene oxide (PO), and cyclohexene oxide (CHO) were performed by both random polymerization and block polymerization to synthesize the random poly (propylene cyclohexene carbonate) (PPCHC), di-block polymers of poly (propylene carbonate–cyclohexyl carbonate) (PPC-PCHC), and tri-block polymers of poly (cyclohexyl carbonate–propylene carbonate–cyclohexyl carbonate) (PCHC-PPC-PCHC). The kinetics of the thermal degradation of the terpolymers was investigated by the multiple heating rate method (Kissinger-Akahira-Sunose (KAS) method), the single heating rate method (Coats-Redfern method), and the Isoconversional kinetic analysis method proposed by Vyazovkin with the data from thermogravimetric analysis under dynamic conditions. The values of ln k vs. T^−1^ for the thermal decomposition of four polymers demonstrate the thermal stability of PPC and PPC-PCHC are poorer than PPCHC and PCHC-PPC-PCHC. In addition, for PPCHC and PCHC-PPC-PCHC, there is an intersection between the two rate constant lines, which means that, for thermal stability of PPCHC, it is more stable than PCHC-PPC-PCHC at the temperature less than 309 °C and less stable when the decomposed temperature is more than 309 °C. Pyrolysis-gas chromatography/mass spectrometry (Py-GC/MS) and thermogravimetric analysis/infrared spectrometry (TG/FTIR) techniques were applied to investigate the thermal degradation behavior of the polymers. The results showed that unzipping was the main degradation mechanism of all polymers so the final pyrolysates were cyclic propylene carbonate and cyclic cyclohexene carbonate. For the block copolymers, the main chain scission reaction first occurs at PC-PC linkages initiating an unzipping reaction of PPC chain and then, at CHC–CHC linkages, initiating an unzipping reaction of the PCHC chain. That is why the T_−5%_ of di-block and tri-block polymers were not much higher than that of PPC while two maximum decomposition temperatures were observed for both the block copolymer and the second one were much higher than that of PPC. For PPCHC, the random arranged bulky cyclohexane groups in the polymer chain can effectively suppress the backbiting process and retard the unzipping reaction. Thus, it exhibited much higher T_−5%_ than that of PPC and block copolymers.

## 1. Introduction

Carbon dioxide (CO_2_) is a nontoxic, nonflammable material that exists naturally in abundance. The use of CO_2_ has attracted increasing interest in recent years and has been considered as an alternative approach to reduce the release of this greenhouse gas [1,2,3,4,5]. One good approach is using CO_2_ to produce biodegradable polymeric materials. In 1969, Inoue et al. first observed that the copolymerization of carbon dioxide with epoxides could form aliphatic polycarbonates [1]. Since then, much work has been done to make CO_2_ copolymerize with other monomers [2,3,4,5]. Poly (propylene carbonate) (PPC) made from carbon dioxide and propylene oxide is the main kind of CO_2_-based copolymer that has been widely investigated [6,7,8,9,10]. In previous work, the high molecular weight alternating PPC was synthesized in very high yield. The PPCs exhibit good biodegradability [11] but show inferior thermal stability due to the flexible carbonate linkage in the backbone. It has also been found that PPC is easily decomposed to cyclic carbonate by the unzipping reaction, which is initiated by the free hydroxyl terminal groups [12,13]. In order to enhance the thermal properties of PPC, a third monomer cyclohexene oxide (CHO) copolymerizing with CO_2_ and PO is considered as a profitable mean [14,15]. However, the thermal degradation kinetics of the terpolymer has not been investigated until now.

Generally, TG analysis is an effective method for studying thermal decomposition kinetics and provides information on a frequency factor, activation energy, and overall reaction order. Due to the insufficient analytical capability of the evolved gas mixture analysis, it is still not possible to provide sufficient information on the mechanism of thermal degradation. Therefore, the direct analysis of gas composition by continuous monitoring with thermogravimetric analysis/Fourier transform infrared spectrometry (TG/FTIR) has attracted more attention in the identification of gaseous products to study the pyrolysis mechanism [16,17,18]. Pyrolysis gas chromatography/mass spectrometry (Py-GC/MS) is widely used to evaluate the thermal decomposition behavior of polymers because of its high sensitivity, rapidity, and effective separation ability of complex compounds containing a pyrolysis product of similar compositions [19,20,21]. Therefore, the combination of TG/FTIR and Py-GC/MS has been widely used to study the thermal decomposition mechanism [22,23,24,25].

In this paper, CHO was introduced to copolymerize with CO_2_ and PO to get the random copolymer, the di-block copolymer, and the tri-block copolymer. The thermal decomposition behaviors of the resultant polymers with different sequence structures are studied by the combination of Py-GC/MS and TG/FTIR techniques. In addition, the thermal degradation kinetic parameters are obtained by using the multiple heating rate method (Kissinger-Akahira-Sunose (KAS) [26,27,28]), the single heating rate method (Coats-Redfern method) [29,30], and the Isoconversional kinetic analysis method proposed by Vyazovkin [31,32,33]. This used the data from thermogravimetric analysis under dynamic conditions such as activation energy E, the pre-exponential factor A, and the rate constant k. They will provide theoretical basis of thermal stability for further application.

## 2. Results and Discussion

### 2.1. Thermal Decomposition Behavior

**Scheme 1:**
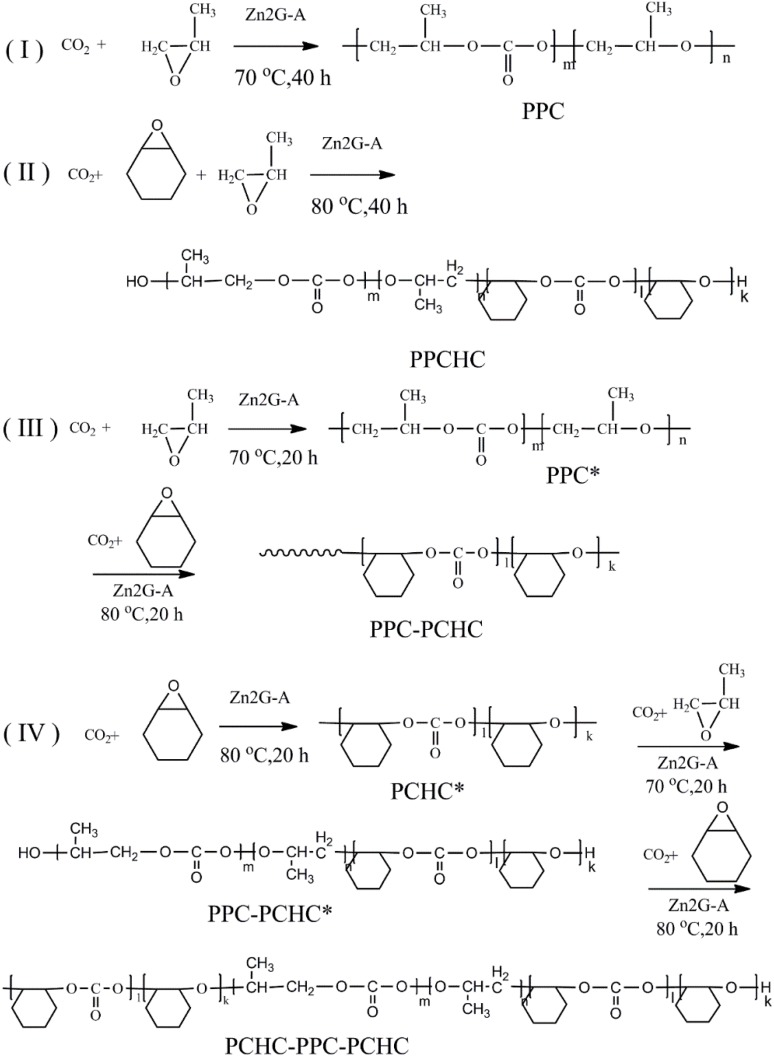
The synthesized procedure of (I) PPC; (II) PPCHC; (III) PPC-PCHC; (IV) PCHC-PPC-PCHC copolymers.

The synthesized copolymers containing polycarbonates and polyesters are terpolymers as Scheme 1. These have proven by our previous paper [15]. ^1^H NMR spectrum of the purified terpolymer shown in Figures S1–S4 demonstrates the formation of ester and carbonate linkages. The proton resonances 1.3 [3H, CH_3_], 4.2 [2H, CH_2_CH], and 5.0 [1H, CH_2_CH] correspond to CH_3_, CH_2_, and CH groups in the polycarbonate sequence. The peaks of 1.7 ppm, 2.1 ppm, and 4.6 ppm representing CH_2_ of M-, O-cycloalkane ring, and CH link to carbonate indicated the cyclohexene carbonate unit in the block copolymer. The basic properties of the resultant polymers are shown in Table 1.

**Figure 1:**
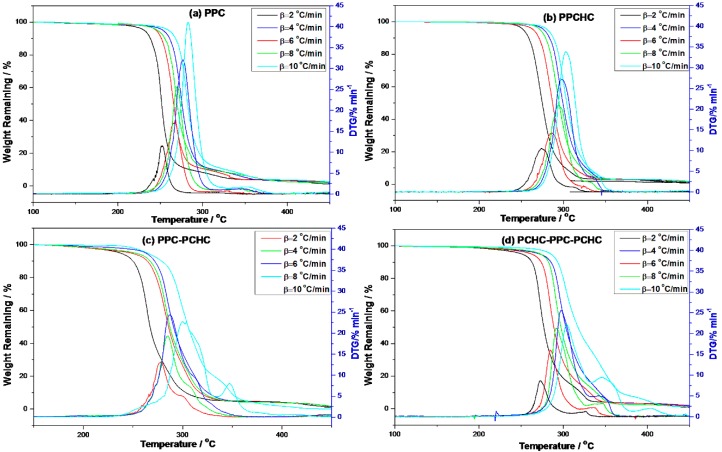
TG-DTG curves of the resultant polymers at different heating rates. (**a**) PPC; (**b**) PPCHC; (**c**) PPC-PCHC; (**d**) PCHC-PPC-PCHC.

In order to study the thermal degradation of the resultant polymers and to conclude the best way to improve the thermal stability of PPC, the non-isothermal kinetics of the thermal degradation of the resultant polymers were investigated with the Kissinger-Akahira-Sunose (KAS) method, the Coats-Redfern method, and the Isoconversional kinetic analysis method proposed by Vyazovkin. TG-DTG curves of the polymers at different heating rates are presented in Figure 1. As the heating rate increased, thermal hysteresis became more and more evident. The thermal decomposition beginning temperature of the polymers also improved and the peak temperature moved to a higher temperature zone. Thermal decomposition of PPC and PPCHC were finished by one step and PPC-PCHC and PCHC-PPC-PCHC were mainly finished by two steps. Decomposition of four polymers at the heating rate 10 °C min^−1^ begin at 245.5 °C, 269.8 °C, 239.8 °C, and 257.2 °C, respectively. The maximum weight loss temperature from DTG curves for four polymers are 278.2 °C, 313.2 °C, 304.2 °C, and 305.8 °C, respectively.

**Figure 2:**
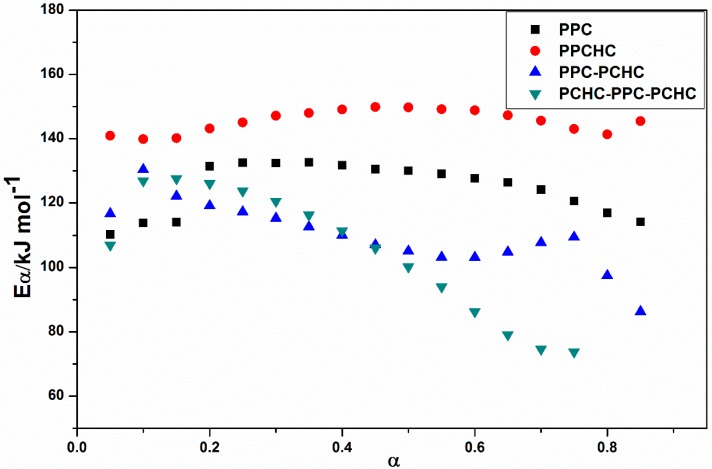
Values of the activation energy estimated by using the KAS method for the thermal decomposition of different polymers.

In addition, the decomposition of four polymers are completed at ~350 °C. The KAS method has been employed to evaluate the activation energies of different polymers during thermal decomposition because of its good adaptability and validity for model-free approaches. At a constant value of conversion rate α, the plots of ln (β/T_α_^2^) versus 1/T_α_. As shown in Figure S5, the plots of ln (β/T_α_^2^) versus 1/T at several heating rates obtain well fitted straight lines whose slopes allow the evaluation of the apparent activation energy. The distribution of the activation energy (Eα) for different polymers is presented in Figure 2. It can be seen that the effective activation energy varies with conversion, which is an indication of a complex mechanism of the decomposition reaction. The process of PPC, PPC-PCHC, and PCHC-PPC-PCHC begins with Eα~110 kJ mol^−1^ and then slightly increases to 130 kJ mol^−1^. In addition, Eα for block polymers PPC-PCHC and PCHC-PPC-PCHC decrease sharply to ~90 kJ mol^−1^ and 75 kJ mol^−1^. For random polymer PPCHC, the Eα remains stable ~140–150 kJ mol^−1^. The average activation energies for PPC, PPCHC, PPC-PCHC, and PCHC-PPC-PCHC are 124.6 ± 6.8 kJ mol^−1^,145.5 ± 3.0 kJ mol^−1^, 109.9 ± 7.6 kJ mol^−1^, and 104.9 ± 16.2 kJ mol^−1^, respectively. The variation tendency tells us that the Eα of the block copolymer PPC-PCHC and PCHC-PPC-PCHC have a similar trend during the process of thermal decomposition while the variation tendency of PPC and PPCHC were relatively stable.

**Figure 3:**
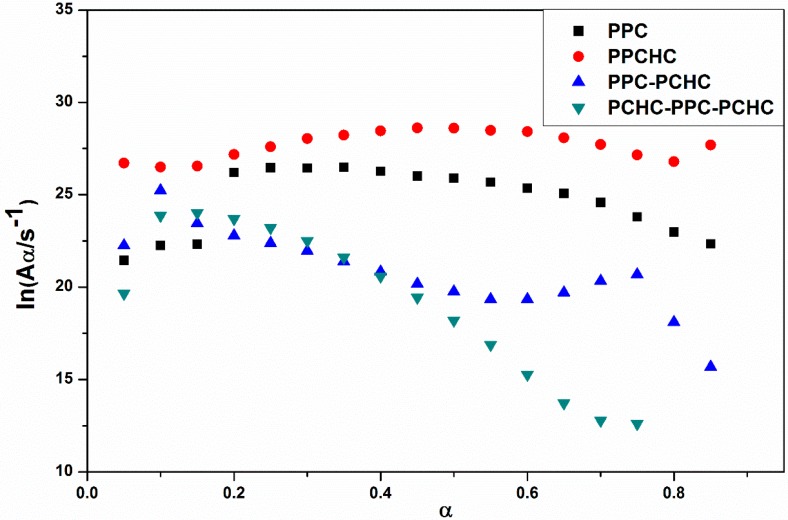
Pre-exponential factor as a function of conversion for the thermal decomposition of different polymers.

**Figure 4:**
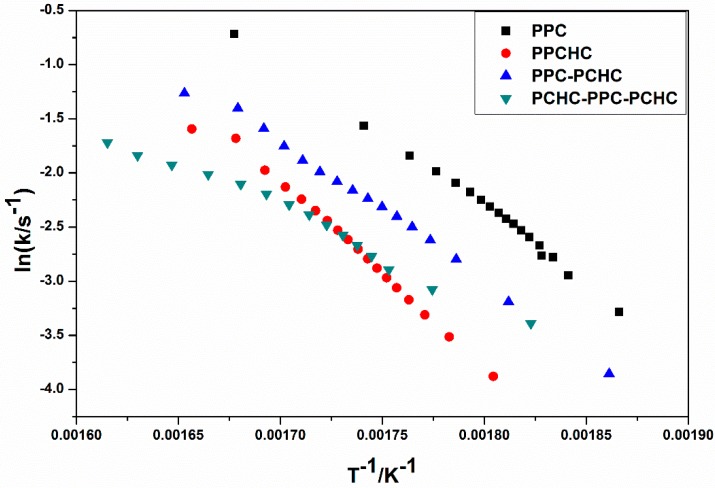
Rate constant as a function of reciprocal temperature for the thermal decomposition of different polymers.

The model-fitting method (Coats-Redfern method) is suitable for the thermal decomposition of polymer during a certain pyrolysis stage in an entire thermal process. The Coats-Redfern method and 15 kinds of reaction models in solid-state reactions are adopted to focus on analyzing the pyrolysis kinetics of the polymers. The obtained kinetic parameters at different heating rates of polymers are presented in Table S1–S4. Then the iso-conversional pre-exponential factor values were evaluated by substituting the values of Eα from the KAS method into the equation of the compensation effect in Equation (3) [31]. The compensation effect parameters a and b were determined by fitting the pairs of ln A_i_ and E_i_ from the Coats-Redfern method using 15 reaction models. The compensation effect parameters a and b were obtained by the average of five heating rates, which is shown in Figure S6. Therefore, the equations of compensation effect are lnA_α_ = −3.23816 + 0.2244E_α_ for PPC, lnA_α_ = −3.5574 + 0.21475E_α_ for PPCHC, lnA_α_ = −2.9405 + 0.21602E_α_ for PPC-PCHC, and lnA_α_ = −3.0112 + 0.211782E_α_ for PCHC-PPC-PCHC. The values of the pre-exponential factor as a function conversion for the thermal decomposition of different polymers are presented in Figure 3. The ln A_α_ vs. α dependence exhibits similar facture as the one determined for the activation energy. The average preexponential factor for PPC, PPCHC, PPC-PCHC, and PCHC-PPC-PCHC are 24.7 ± 1.5 s^−1^, 27.7 ± 0.6 s^−1^, 20.8 ± 1.6 s^−1^, and 19.2 ± 3.4 s^−1^, respectively. Overall, the trend of activation energy values and pre-exponential factor values for decomposition is PCHC-PPC-PCHC < PPC-PCHC < PPC < PPCHC. However, the thermal stability of polymers determined by an increase activation energy values and a decrease pre-exponential factor values. In order to evaluate the overall effect of the activation energy and the pre-exponential factor on the kinetics of the process, the rate constant was calculated by using Equation (4) [32,33]. The values of ln k vs. T^−1^ for the thermal decomposition of four polymers are presented in Figure 4. From the results, decomposition of PPC, PPC-PCHC show higher values of the rate constant, so the thermal stability of PPC and PPC-PCHC are poorer than PPCHC and PCHC-PPC-PCHC. In addition, for PPCHC and PCHC-PPC-PCHC, there is an intersection between the two rate constant lines, which means that, for thermal stability, PPCHC is more stable than PCHC-PPC-PCHC at temperatures less than 309 °C and less stable when the temperature is more than 309 °C. The enhanced thermal stability of PPCHC is associated with an increase in the activation energy and the block polymer PPC-PCHC and PCHC-PPC-PCHC is mostly associated with a decrease in the pre-exponential factor. Above all, the random copolymerization of CHO, PO, and CO_2_ is a better way to improve the thermal stability of PPC. We began with the idea that the tri-block polymer PCHC-PPC-PCHC had the same structure like SBS, which means its thermal stability was better than others. However, the results were not the same as predicted. In order to explain this, we studied the thermal degradation mechanisms of the polymers, which is discussed in the following part.

### 2.2. Study the Thermal Decomposition Behavior Using Py-GC/MS and TG/FTIR

Thermal degradation kinetic parameters of the polymers were calculated by using thermogravimetric analysis. However, it could not provide enough information to analyze the thermal degradation mechanism. With the help of TG/IR technique, we can follow the dynamic process of the polymers decomposition. FTIR spectra (Figure S7) in three-dimensions for the decomposing dynamic process of polymers were obtained via this technique. The three-dimensional FTIR spectra of the pyrolysates derived from PPCHC, PPC-PCHC, and PCHC-PPC-PCHC are similar and the main IR absorbing peaks appear at 1109 cm^−1^, 1863 cm^−1^, and 2985 cm^−1^, which indicates that the main pyrolysates of the block polymers are all cyclic carbonates.

**Figure 5:**
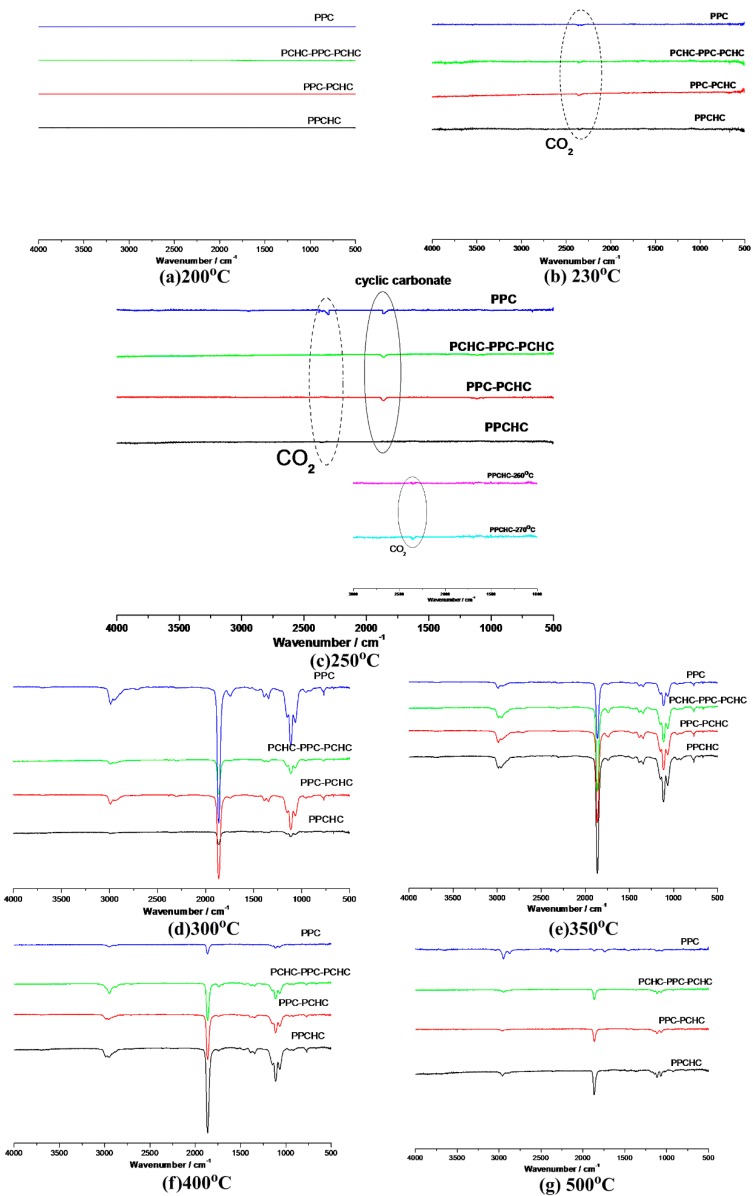
FTIR spectra of pyrolysates at (**a**) 200 °C; (**b**) 230 °C; (**c**)250 °C; (**d**) 300 °C; (**e**) 350 °C, (**f**) 400 °C, and (**g**) 500 °C.

**Figure 6:**
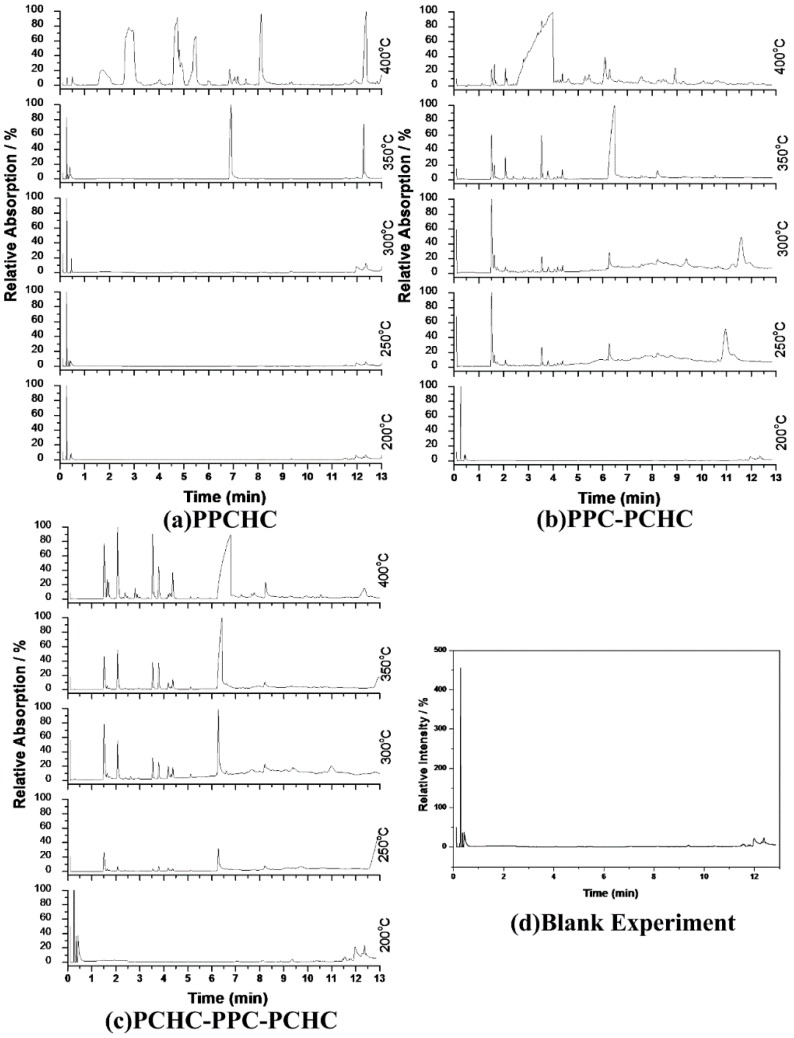
Chromatogram of pyrolysis products by Py-GC/MS at different temperatures :(**a**) PPCHC; (**b**)PPC-PCHC; (**c**)PCHC-PPC-PCHC; (**d**) Blank Experiment.

According to previous research [34,35,36,37], the thermal decomposition behavior of PPC obeys two kinds of mechanism including the main chain scission reaction and the unzipping reaction. The unzipping reaction involves the backbiting of the terminal hydroxyl groups at the carbon of carbonate linkage leading to the formation of cyclic carbonate. From the FTIR spectra of pyrolysates at different temperatures shown in Figure 5, it can be seen that the peak stands for carbon dioxide is observed at 230 °C while the peaks stand for cyclic carbonates are observed at 250 °C for PPC, PPC-PCHC, and PCHC-PPC-PCHC and higher than 270 °C for PPCHC, which indicates the main chain scission reaction and the unzipping reaction during the decomposition process first occuring in PC-PC segments. In addition, the onset unzipping reaction temperature of the random polymer PPCHC is higher than that of PPC and block polymers. Comparing the intensity of the IR absorption peaks of the pyrolysates at a different temperature, we can see that large scale evolution of cyclic carbonates happens at a relatively lower temperature (about 300 °C) for PPC and higher temperature (about 350–400 °C) for PPCHC. While for the block copolymers, large scale evolution of cyclic carbonates happens at a wider temperature range (300–400 °C). It is supposed that the random polymer PPCHC probably has a large content of PC-CHC or CHC-PC linkages in the terpolymer, which means the steric hindrance of bulky cyclohexane groups can suppress the backbiting process and retard the unzipping reaction. Thus, the thermal stability of PPCHC is much better than PPC. While, for the block copolymers, the PC-PC block and CHC-CHC block exhibit similar thermal stability as PPC and PCHC, respectively, so the temperature range of large scale evolution of cyclic carbonate was wide and two maximum decomposition temperatures were observed, as shown in Table 1. The above supposition was further confirmed by Py-GC/MS, which provide a way to measure the thermal decomposition reactions in each stage by analyzing mixture-evolved gases. Based on the results of TG analysis, the pyrolysis temperatures were set at 200 °C, 250 °C, 300 °C, 350 °C, and 400 °C to obtain the formation curves of pyrolysates to analyze the degradation mechanisms. The chromatograms of pyrolysis products at different temperatures and their identifications are shown in Figure 6 and Table 2.

The pyrolysates of random copolymer PPCHC was not detected out at 300 °C and, at 350 °C, the pyrolysates are cyclic propylene carbonate (6.0–6.75 min) and cyclic cyclohexene carbonate (12.25–12.5 min). These phenomena confirms the large content of PC-CHC or CHC-PC linkages in the polymer chain. Due to the alternating PC and CHC main chain structure, the backbiting is suppressed by steric hindrance results in a retarded unzipping reaction and, once the unzipping reaction starts, the pyrolysates cyclic propylene carbonate and cyclic cyclohexene carbonate are produced at the same time.

**Scheme 2:**
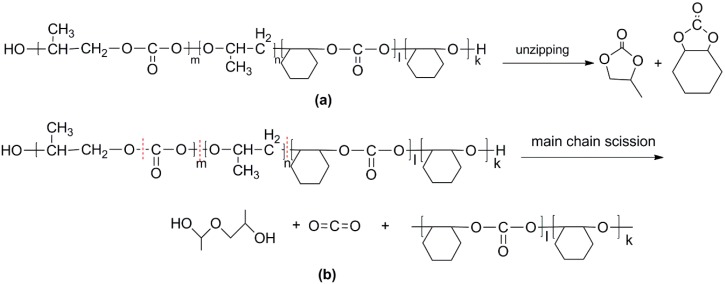
Possible decomposition pathway of the polymers. (**a**) Unzipping of the polymer backbone and (**b**) main chain scission.

For the di-block and tri-block copolymers, small peaks representing CO_2_ (1.5–2.0 min) and cyclic propylene carbonate (6.0–6.75 min) appear when being pyrolyzed at 250 °C. The peak of cyclic cyclohexene carbonate appears at a pyrolysis temperature as high as 350 °C, which means that the main chain scission reaction first occurs at PC-PC linkages. This initiates the unzipping reaction of PC-PC linkages and then, at CHC-CHC linkages, initiates the unzipping reaction of the PCHC chain. In addition, this can explain why the degradation activation energies of the block polymers are lower at first and get higher with the decomposition conversion increasing. Based on above results and analysis, the possible decomposition pathways are given as Scheme 2. For the random copolymer PPCHC, the thermal decomposition behavior obeys the unzipping reaction mostly. In addition, for the block copolymers of PPC-PCHC and PCHC-PPC-PCHC, the thermal decomposition behavior obeys two kinds of mechanism including the main chain scission reaction and the unzipping reaction.

## 3. Materials and Methods

### 3.1. Materials

Carbon dioxide of a purity of 99.99% was used without further treatment. PO of a purity of 99.5% and CHO of a purity of 95.0% were refluxed over CaH_2_ for 4 h and 24 h, respectively, and then distilled under dry nitrogen gas. Prior to use, they were stored over 4-Å molecular sieves. Other solvents and reagents such as ethanol and chloroform were of analytical grade and used without further purification.

Supported multi-component zinc dicarboxylate catalyst (Zn2G) was prepared according to previous work [38]. The catalyst was white powder with the Zn content of 11.6 wt %.

### 3.2. Preparation of Random Terpolymers and Block Copolymers

The di-block PPC-PCHC copolymerization was carried out using ‘one pot, two steps’ method in a 500 mL stainless steel autoclave equipped with a mechanical stirrer [15]. A multi-component catalyst (Zn2G) was introduced into the autoclave and the autoclave with the catalyst inside was dried for 24 h under vacuum at 80 °C and then cooled down to room temperature. Then the purified PO was immediately injected into the autoclave. The autoclave was pressurized to 5.2 MPa via a CO_2_ cylinder and heated at 70 °C for 20 h. Following the evacuation of CO_2_ and unreacted PO, CHO was introduced into the autoclave under an inert atmosphere. The autoclave was re-pressurized with 5.2 MPa of CO_2_ and the reaction was performed at 80 °C for another 20 h. Then, the pressure in the autoclave was reduced to atmosphere to terminate the block copolymerization. Similarly, the tri-block polymer PCHC-PPC-PCHC was synthesized by using the ‘one pot, three steps’ method. The first step was the copolymerization of CO_2_ with CHO, which is followed by the copolymerization of CO_2_ with PO, and then the copolymerization of CO_2_ with CHO again [15]. The random copolymer poly (propylene cyclohexene carbonate) (PPCHC) was prepared by terpolymerization of CO_2_, PO, and CHO [14]. For the first 30 h, the temperature was set at 70 °C and, for the next 10 h, it was raised to 80 °C. PPC was made from PO and CO_2_ while PCHC was a copolymer of CHO and CO_2_. The resulting copolymers were separately dissolved in a proper volume of chloroform and 15 mL dilute HCl (5 wt %) was added to extract the catalyst residual from the product solution. The organic layer was then washed with distilled water three times. The viscous solution was concentrated to a proper concentration by using a rotary evaporator. Lastly, they were precipitated by being poured into vigorously stirred ethanol. The as-made copolymers were filtered and dried under vacuum at a temperature of 120 °C until a constant weight was obtained.

### 3.3. Determination of the Composition and the Molecular Weight of Polymers

^1^H-NMR spectra of the block copolymer at room temperature using tetramethylsilane as an internal standard and D-chloroform (CDCl_3_) as a solvent were recorded on a Bruker DRX-400 NMR spectrometer (Karlsruhe, Germany). Molecular weight (M_w_ and M_n_) of the resultant polymer product was measured by a gel permeation chromatography (GPC) system (Waters 515 HPLC Pump, Waters 2414 detector) (Milford, MA, USA) with a set of three columns (Waters Styragel 500, 10,000, and 100,000 Å) and chloroform (HPLC grade) as eluent. The GPC system was calibrated by a series of polystyrene standards with polydisperisties of 1.02.

### 3.4. Py-GC/MS Measurement

The Py-GC/MS measurements were carried out using a PYR-2A micro-tube furnace pyrolyser (Shimadzu-PYR2A) (Kyoto, Japan) coupled to an HP 6890 gas chromatograph linking to an HP 5973 quadruple mass spectrometer. The sample was heated in the PYR-2A compact in an oxygen-free furnace. Sample aliquots of about 1.00 mg were pyrolyzed by using a platinum coil attachment. This furnace’s temperatures were set at 200 °C, 250 °C, 300 °C, 350 °C, and 400 °C, respectively. A quartz capillary column (SE-30; 15 m × 0.2 mm) (Kyoto, Japan) was used in the GC. The column temperature was initially held at 45 °C for 1 min and then programmed to 250 °C at a heating rate of 10 °C/min. The GC/MS interface was set at 280 °C. The pyrolysed products of polymers were directly injected and separated by gas chromatography using helium as an eluent gas and then characterized by the mass spectrometer. The mass spectra were recorded under electron impact ionization energy at 70 eV. The MS detector scanned from 29 to 350 m/z at a scan rate of 1.8 scan/s.

### 3.5. TG/FTIR Measurement

Thermogravimetric analysis (TGA) measurements were performed in a PerkinElmer Pyris Diamond TG/DTA analyzer (Waltham, MA, USA) under a protective nitrogen atmosphere. The temperature ranged from 50–500 °C with a heating rate of 2, 4, 6, 8, 10 °C/min, respectively. TG/IR analysis was performed with a TG/IR system, which combined with a PerkinElmer Pyris Diamond TG/DTA analyzer and a PerkinElmer Spectrum 100 FTIR spectrometer (Waltham, MA, USA). Samples of about 10 mg were pyrolysed in the TG analyzer and the evolved gases were led to the FTIR spectrometer directly through a connected heated gas line to obtain three dimensional FTIR spectra. The flow rate of N_2_ is 10 mL/min. The aluminum pans are used for the samples. The temperature of the heated transfer line is 200 °C. The heating rate for taking TGA/FTIR spectra is 10 °C /min. The operating conditions of the FTIR had a frequency range of 4000–400 cm^−1^, a resolution of 2.0 cm^−1^, and a scan rate of 1.0 scan/s.

### 3.6. Thermal Decomposition Kinetics

The “International Confederation for Thermal Analysis and Calorimetry (ICTAC)” committee recommended that utilizing multiple heating rate programs leads to more reliable kinetic parameters with respect to the single heating rate program [31]. The Kissinger-Akahira-Sunose (KAS) method is an integral method by which the E can be obtained through the conversion values of reactant [26,27,28], which is represented by Equation (1) below.

(1) $$ln\frac{\beta }{T^{2}}=ln\left(\frac{AE}{Rg\left(\alpha \right)}\right)-\frac{E}{RT}$$

Definitions of all the variables and parameters are the same with previous equations. Since the value of $\ln\left(\frac{AE}{Rg\left(\alpha \right)}\right)$ is approximately constant when the values of α are the same at different β, the plot $\ln\frac{\beta }{T^{2}}$ versus 1/T is approximately linear. Thus, by plotting $\ln\frac{\beta }{T^{2}}$ against 1/T at certain conversion rates, the slope $-\frac{E}{RT}$ is calculated E.

The parameters of the compensation effect of the polymers’ pyrolysis were obtained by the Coats-Redfern method. Fifteen kinds of frequently used reaction mathematical models are substituted into the Coats-Redfern equation. The Coats-Redfern equation is expressed by Equation (2) [29,30].

(2) $$ln\frac{g\left(\alpha \right)}{T^{2}}=ln\frac{AR}{\beta E}-\frac{E}{RT}$$

Substituting g(α) into Equation (2) and plotting $\ln\frac{g\left(\alpha \right)}{T^{2}}$ versus 1/T, E and lnA of the different mathematical models can be calculated based on the slope (-E/R) and intercept ($ln\frac{AR}{\beta E}$).

The iso-conversional pre-exponential factor lnA and rate constant *lnk* are obtained by using the iso-conversional kinetic analysis method proposed by Vyazovkin [31,32,33]. The ln A is determined by the reaction feature of the reactant and it is independent of temperature. Therefore, the calculation is important for understanding the reaction feature. Since the compensation effect exists in E and A, Equation (3) is usually used to calculate ln A [31].

(3) lnA=aE+b

The compensation effect parameters a and b were determined by fitting the pairs of lnAi and Ei by 15 different models substituting into the Coats-Redfern method at each heating rate. Then, lnA can be determined for every conversion α by substituting the respective values of E from the KAS method into Equation (3) to obtain a dependence of lnA on α. LnA_α_ on α and Eα on α data were converted into the Arrhenius plot. Therefore, the rate constant for the thermal decomposition can be evaluated as Equation (4) [32,33].

(4) $$lnk\left(T_{\alpha }\right)=lnA_{\alpha }-\frac{E_{\alpha }}{RT_{\alpha }}$$

## 4. Conclusions

Thermal decomposition behaviors and degradation kinetic parameters of terpolymers with different sequence structures derived from CO_2_, PO, and CHO were studied by the combination of Py-GC/MS and TG/IR techniques. In addition, the thermal degradation kinetic parameters were calculated by the Kissinger-Akahira-Sunose (KAS) method, the Coats-Redfern method, and the iso-conversional kinetic analysis method proposed by Vyazovkin with the data from thermogravimetric analysis under dynamic conditions. The average degradation activation energies of the polymers are PCHC-PPC-PCHC (104.9 ± 16.2 kJ mol^−1^) < PPC-PCHC (109.9 ± 7.6 kJ mol^−1^ L) < PPC (124.6 ± 6.8 kJ/mol)) < PPCHC (145.5 ± 3.0 kJ mol^−1^). The average pre-exponential factor ln A_α_ for PPC, PPCHC, PPC-PCHC, and PCHC-PPC-PCHC are 24.7 ± 1.5 s^−1^, 27.7 ± 0.6 s^−1^, 20.8 ± 1.6 s^−1^, and 19.2 ± 3.4 s^−1^, respectively. The rate constant values ln k vs. T^−1^ for the thermal decomposition of four polymers demonstrated that the thermal stability of PPC and PPC-PCHC are poorer than PPCHC and PCHC-PPC-PCHC and for PPCHC and PCHC-PPC-PCHC. There is an intersection between the two rate constant lines, which means that, for thermal stability, PPCHC is more stable than PCHC-PPC-PCHC at temperatures less than 309 °C and less stable when the temperature is more than 309 °C. The thermal degradation mechanism of the polymers was elucidated by IR-TG and Py-GC/MS techniques to be the main chain scissor reaction followed by the unzipping reaction. Due to large content of PC-CHC or CHC-PC linkages in the PPCHC, the steric hindrance of bulky cyclohexane groups restricted the unzipping reaction to some extent. The random copolymer showed one step decomposition with a T_−5%_ and T_max_ as high as 281.0 °C and 313.4 °C, respectively. For the block polymers, the chain scission and unzipping reaction occurred first at the PPC block and then at the PCHC block. The CHC-CHC linkages could not restrict the PC-PCs unzipping reaction well, so the T_−5%_ of di-block and tri-block polymers are 261.2 °C and 275.2 °C, respectively, while two maximum decomposition temperatures were observed at 304.2–342.7 °C, and 305.8–345.3 °C, respectively. Lastly, we can conclude that random copolymerization of CHO, PO, and CO_2_ is a better way to improve the thermal stability of PPC than block copolymerization.

## Figures and Tables

**Table 1:** The properties of the resultant polymers.

Copolymer	Mn/Mw/PI ^a^	T_−5%_/T_max_ (°C) ^c^	Composition (Molar Fraction %) ^b^			
			$f_{CO2}$	$f_{PC}$	$f_{CHC}$	$f_{PE}$
**PPC**	2.17 × 10^5^/3.78 × 10^5^/1.74	255.7/278.2	48.9	48.9	-	2.2
**PPCHC**	2.02 × 10^5^/7.13 × 10^5^/3.50	281.0/313.4	47.9	36.2	11.7	4.2
**PPC-PCHC**	2.97 × 10^5^/7.35 × 10^5^/2.47	261.2/304.2, 342.7	48.0	35.1	12.9	4.0
**PCHC-PPC-PCHC**	2.74 × 10^5^/7.88 × 10^5^/2.87	275.2/305.8, 345.3	47.7	34.1	13.6	4.6

^a^ Molecular weight was determined by GPC. ^b^ Determined by ^1^H NMR spectroscopy. (see ESI Figure A1–A4) $f_{PC}=A_{4.2}/\left[\left(A_{4.2}+A_{4.6}\right)\times 2+0.8\times A_{3.5}\right]$, $f_{CHC}=A_{4.6}/\left[\left(A_{4.2}+A_{4.6}\right)\times 2+0.8\times A_{3.5}\right]$, $f_{CO2}=A_{4.2}+A_{4.6}/\left[\left(A_{4.2}+A_{4.6}\right)\times 2+0.8\times A_{3.5}\right]$, $f_{PE}=0.8\times A_{3.5}/\left[\left(A_{4.2}+A_{4.6}\right)\times 2+0.8\times A_{3.5}\right]$. ^c^ The heating rate is 10 °C/min.

**Table 2:** Identification of pyrolysates in Py-GC/MS.

Compound	Retention Time (min)	Compound	Retention Time (min)
		**CO_2_**	1.5–2 min
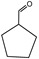	4.0–4.5 min	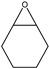	4.5–5.0 min
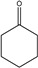	5.0–5.5 min	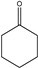	6.0–6.75 min
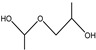	7.5–8.0 min	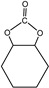	12.25–12.5 min

## Supplementary Materials

Supplementary materials can be found at http://www.mdpi.com/1422-0067/19/12/3723/s1.

## Abbreviations

TG/FTIR	Thermogravimetric analysis/ Fourier transform infrared spectrometry
Py-GC/MS	Pyrolysis-gas chromatography/mass spectrometry
PPC	poly (propylene carbonate)
PPCHC	Poly (propylene cyclohexene carbonate)
PPC-PCHC	Poly (propylene carbonate–cyclohexyl carbonate)
PCHC-PPC-PCHC	Poly (cyclohexyl carbonate–propylene carbonate–cyclohexyl carbonate)
