# Supplementary material for: Study on Thermal Decomposition Behaviors of Terpolymers of Carbon Dioxide, Propylene Oxide, and Cyclohexene Oxide
Source: Int J Mol Sci. 2018 Nov 23;19(12):3723. doi: 10.3390/ijms19123723 (PMC6320886; doi:10.3390/ijms19123723)
Supplement: Supplementary file 1 [file ijms-19-03723-s001.pdf]

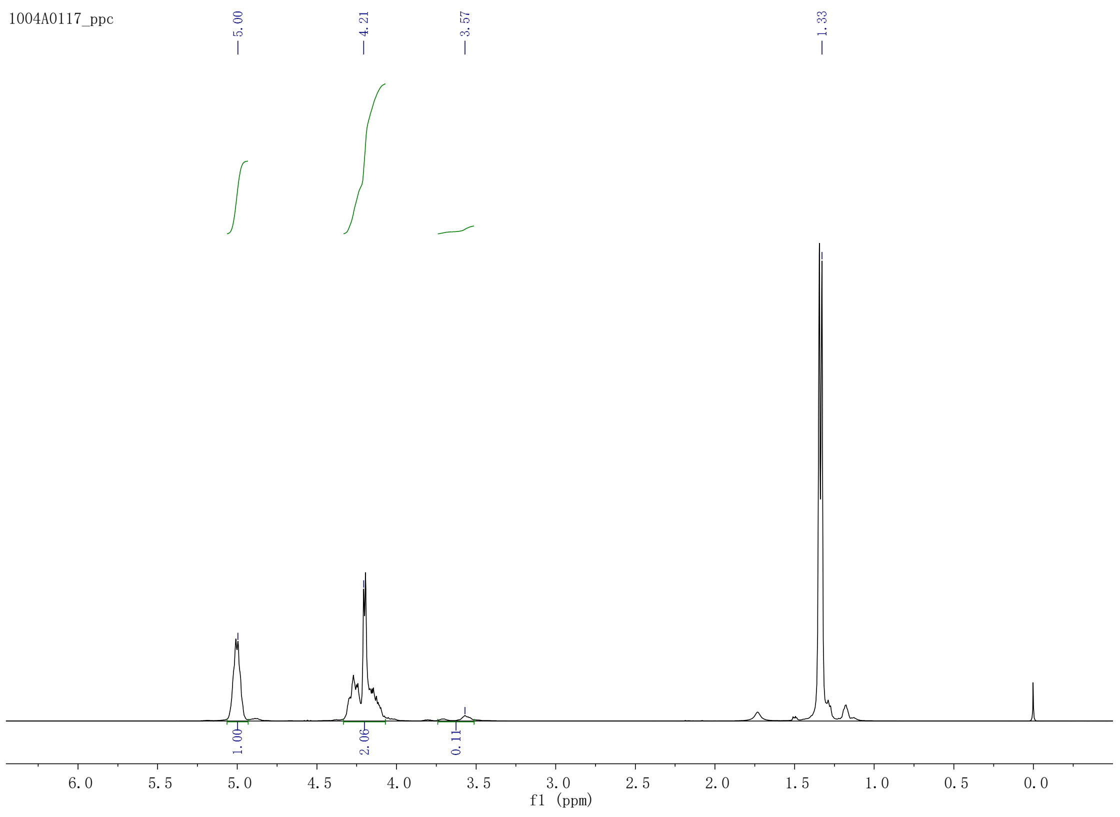

Figure S1. ^{1}H-NMR spectrum of PPC

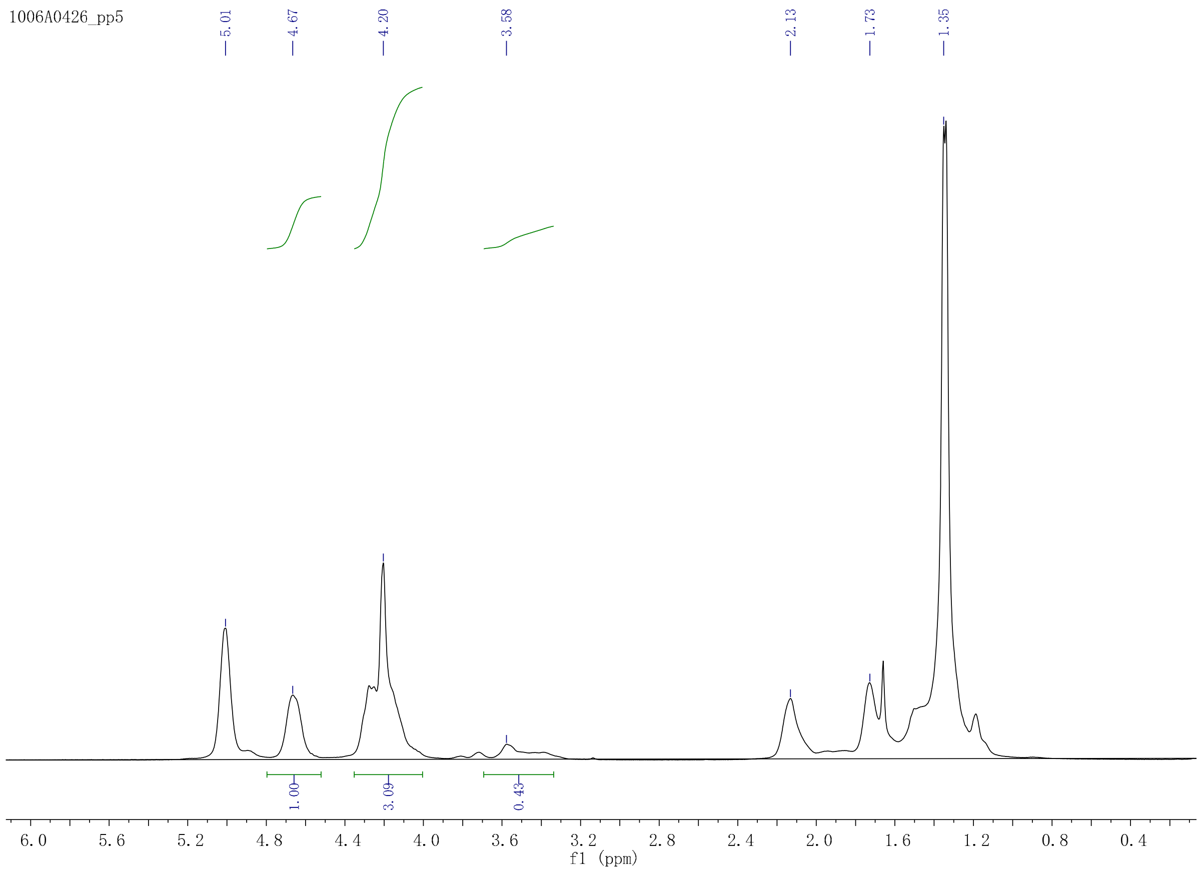

Figure S2. ^{1}H-NMR spectrum of PPCHC

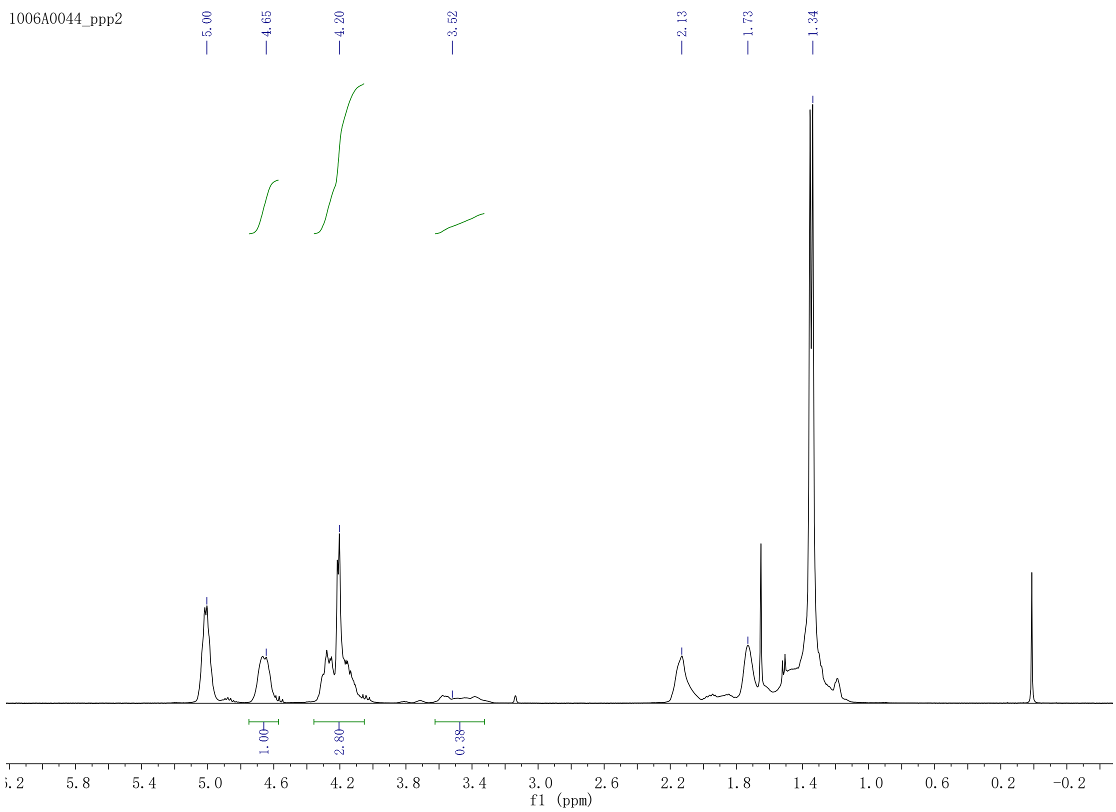

Figure S3. ^{1}H-NMR spectrum of PPC-PCHC

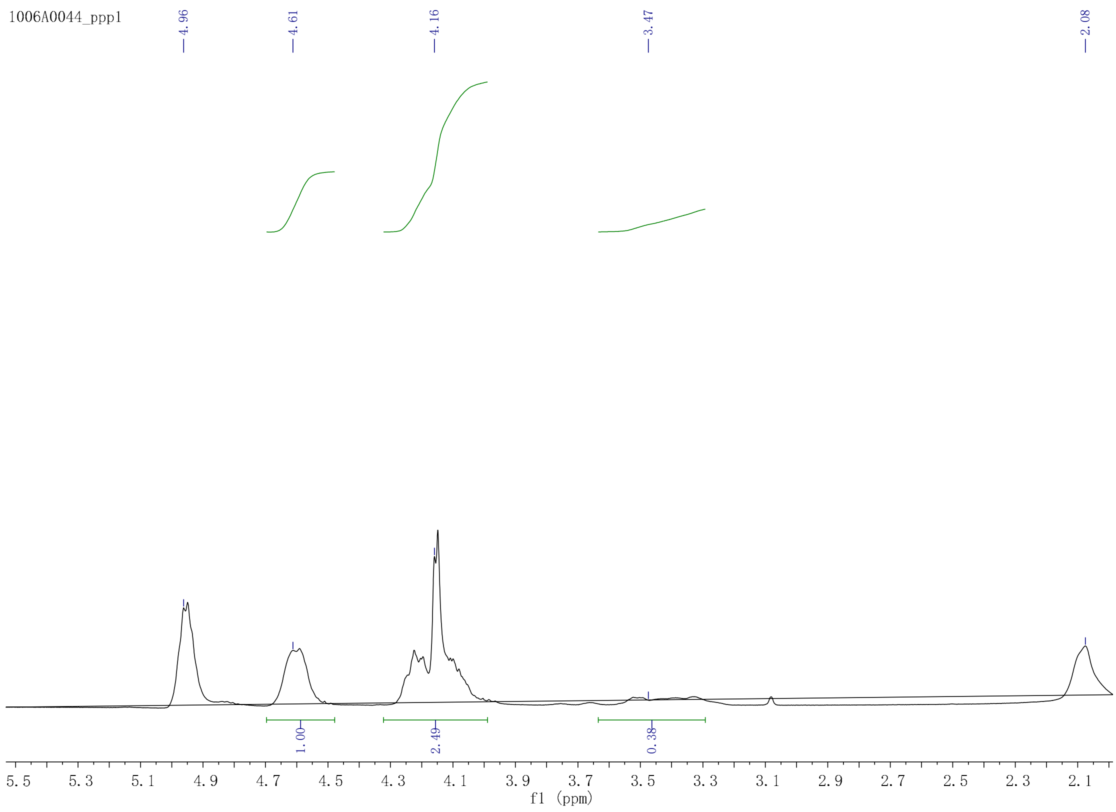

Figure S4. ^{1}H-NMR spectrum of PCHC-PPC-PCHC

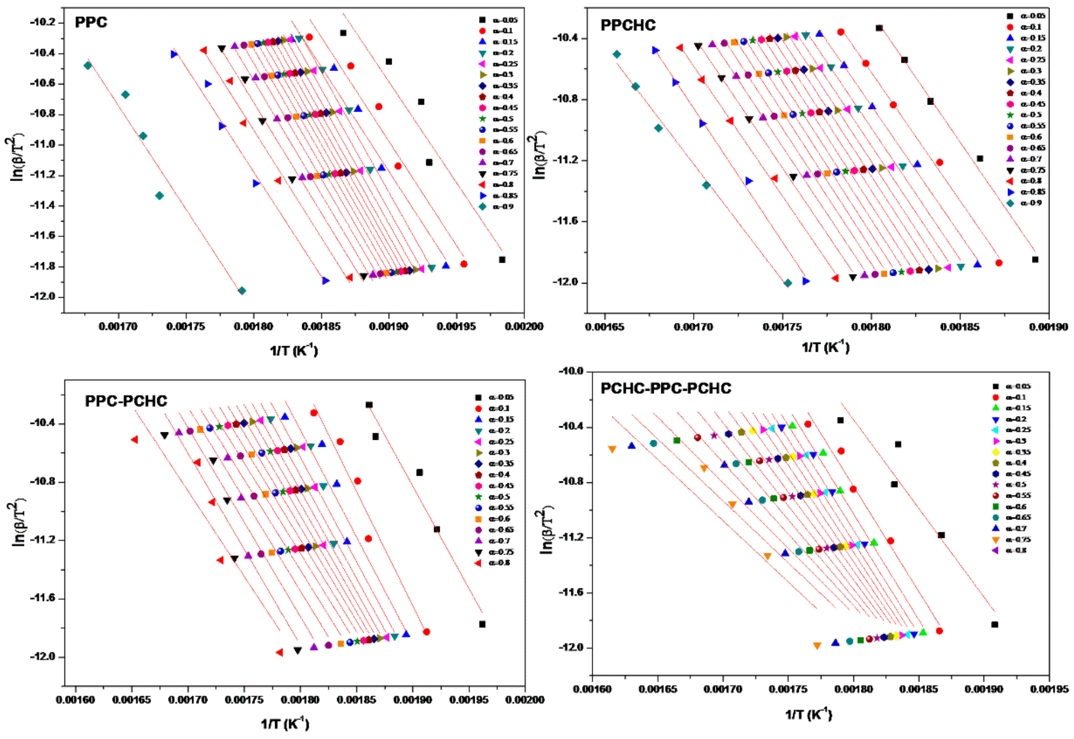

Figure.S5 Global kinetic plots of different polymers for KAS iso-conversional method

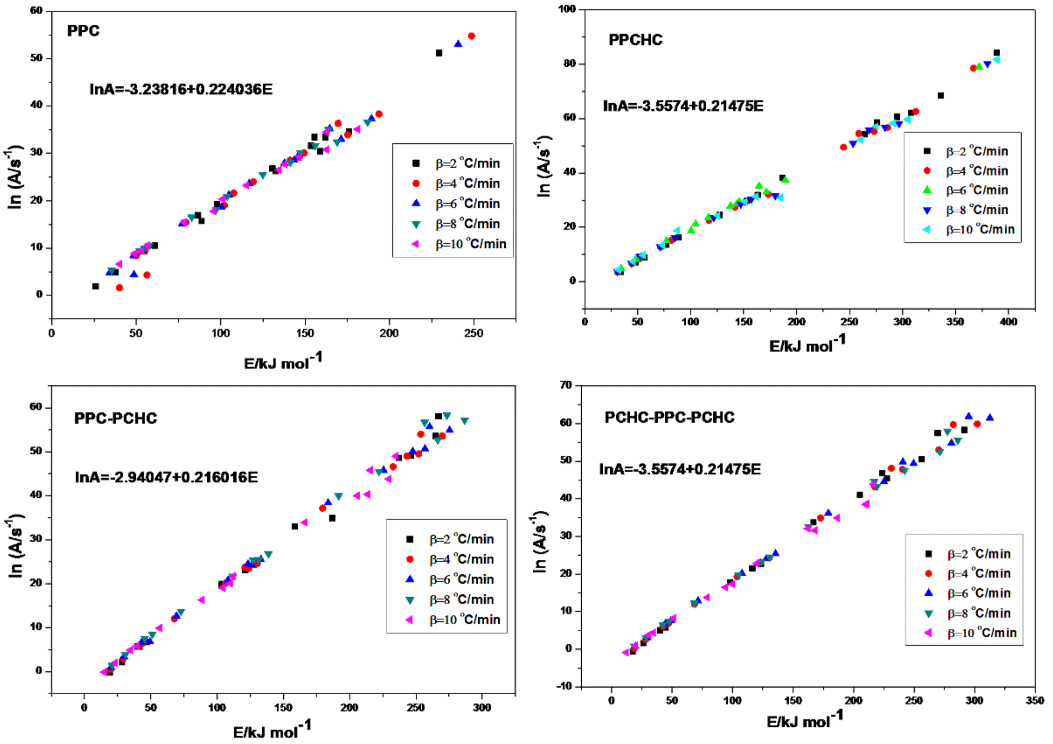

Figure.S6 Kinetics compensation effect of different polymers by fitting pairs of lnAi and Ei by 15 different models at each heating rate

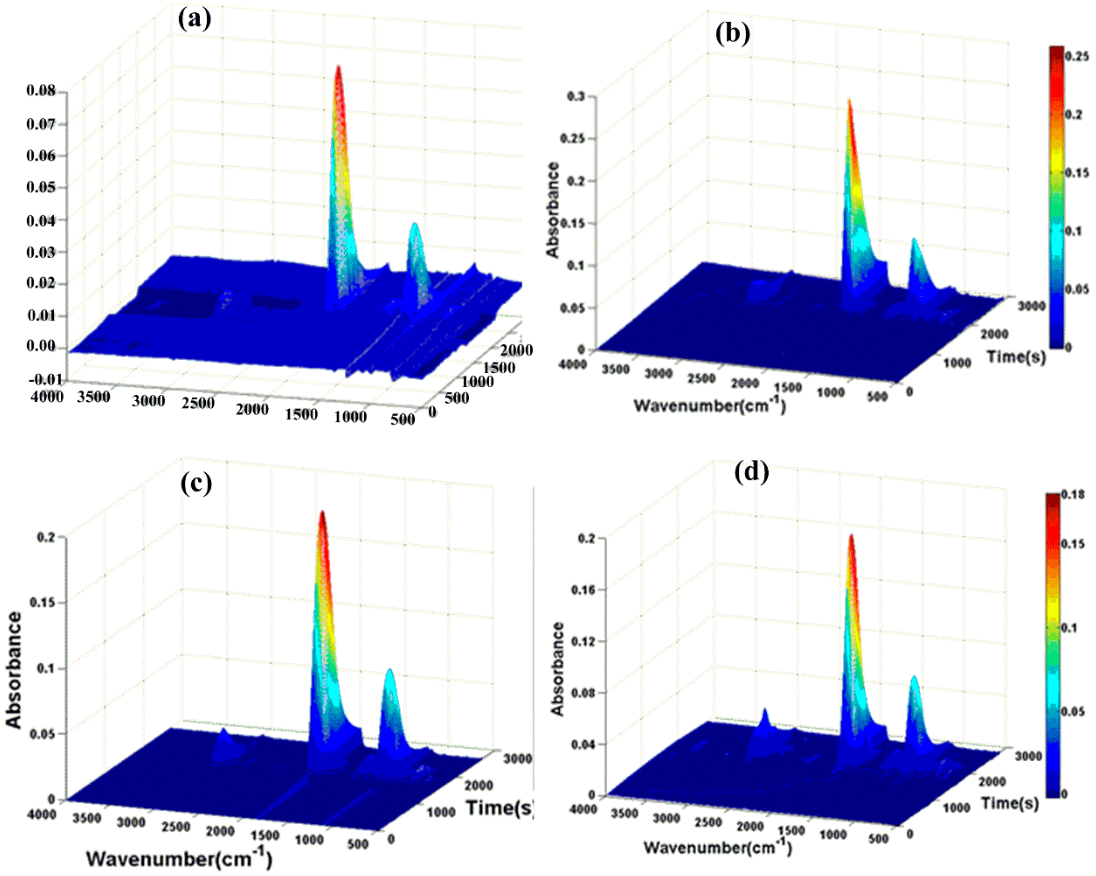

Figure.S7 Three-dimensional FTIR spectra
(a) PPC (b) PPCHC (c) PPC-PCHC (d) PCHC-PPC-PCHC

Table A1 Kinetic parameter of PPC thermal decomposition by Coats-Redfern method

Method No.	β=2 K min^{-1}			β=2 K min^{-1}			β=2 K min^{-1}			β=2 K min^{-1}			β=2 K min^{-1}		
	E /kJ mol^{-1}	ln A	R^2	E /kJ mol^{-1}	ln A		E /kJ mol^{-1}	ln A	R^2	E /kJ mol^{-1}	ln A		E /kJ mol^{-1}	ln A	R^2
F1	97.9	19.3	0.5717	107.7	21.6	0.6332	104.9	21.2	0.6587	103.8	20.9	0.6189	101.1	20.3	0.5554
F2	155.5	33.4	0.7283	169.6	36.3	0.7877	164.6	35.2	0.8106	163.9	35.0	0.7747	162.7	34.5	0.7283
F3	229.3	51.2	0.8142	248.6	54.8	0.8686	240.7	53.0	0.8878	163.9	35.0	0.7747	162.7	34.5	0.7283
D1	132.6	26.2	0.4411	146.6	29.2	0.4938	143.6	28.6	0.5192	141.3	28.1	0.4822	134.6	26.5	0.4108
D2	88.7	15.7	0.4062	102.1	19.0	0.4945	100.4	18.7	0.5153	97.3	18.1	0.4725	95.8	17.8	0.4785
D3	176.1	34.6	0.5406	193.8	38.3	0.5979	189.3	37.3	0.6237	187.0	36.6	0.5854	181.1	35.1	0.5197
D4	158.9	30.4	0.5026	175.2	33.9	0.5586	171.3	33.0	0.5844	169.0	32.4	0.5463	162.7	30.8	0.4782
A2	86.4	16.9	0.7924	79.4	15.4	0.7635	77.2	15.1	0.8068	82.8	16.5	0.8003	78.2	15.4	0.6714
A3	54.7	9.3	0.7737	49.9	8.4	0.7403	48.5	8.4	0.7860	52.1	9.4	0.7803	49.0	8.8	0.6420
R1	130.6	26.8	0.7109	119.4	24.0	0.6724	116.8	23.6	0.7150	125.0	25.5	0.7110	115.4	23.2	0.5667
R2	153.5	31.6	0.7612	141.1	28.5	0.7292	137.8	27.9	0.7715	147.3	30.0	0.7658	137.9	27.7	0.6321
R3	162.2	33.4	0.7776	149.5	30.1	0.7479	145.9	29.4	0.7899	155.9	31.6	0.7837	146.6	29.3	0.6543
P2	60.9	10.5	0.6797	55.2	9.4	0.6345	53.8	9.4	0.6787	57.9	10.5	0.6766	53.0	9.4	0.5218
P3	37.7	4.9	0.9118	56.4	4.3	0.5912	48.6	4.4	0.6365	35.5	5.3	0.6370	56.6	10.5	0.7427
P4	26.0	1.9	0.9031	40.1	1.6	0.5419	34.2	4.8	0.8387	54.7	9.9	0.8885	40.1	6.6	0.7199

Table A2 Kinetic parameter of PPCHC thermal decomposition by Coats-Redfern method

Method No.	β=2 K min^{-1}			β=2 K min^{-1}			β=2 K min^{-1}			β=2 K min^{-1}			β=2 K min^{-1}		
	E /kJ mol^{-1}	ln A	R^2	E /kJ mol^{-1}	ln A		E /kJ mol^{-1}	ln A	R^2	E /kJ mol^{-1}	ln A		E /kJ mol^{-1}	ln A	R^2
F1	186.4	38.2	0.8960	173.3	22.1	0.6332	104.9	21.2	0.6587	179.9	21.5	0.6189	184.8	20.9	0.5554
F2	275.9	58.7	0.8682	258.7	54.5	0.9612	164.6	35.2	0.8106	268.2	55.9	0.9670	275.0	57.1	0.9696
F3	388.9	84.3	0.9916	366.9	78.6	0.9896	372.1	79.0	0.99118	380.1	80.3	0.9942	389.2	81.8	0.9945
D1	264.4	54.3	0.7795	244.2	49.5	0.7426	143.6	28.6	0.5192	253.3	50.9	0.7479	260.6	52.2	0.7561
D2	294.9	60.6	0.8167	273.1	55.3	0.7823	100.4	18.7	0.5153	283.3	56.8	0.7884	291.4	58.3	0.7958
D3	336.2	68.5	0.8624	312.4	62.6	0.8320	189.3	37.3	0.6237	283.3	56.8	0.7884	291.4	58.3	0.7958
D4	308.4	62.2	0.8330	285.9	56.7	0.7999	171.3	33.0	0.5844	296.6	58.2	0.8062	304.9	59.7	0.8133
A2	88.6	16.4	0.8856	81.9	15.2	0.8541	77.2	15.1	0.8068	85.1	16.2	0.8621	87.5	16.8	0.8686
A3	56.0	8.9	0.8737	51.5	8.3	0.8379	48.5	8.4	0.7860	52.1	9.4	0.7803	55.1	9.7	0.8543
R1	127.6	24.6	0.7665	117.4	22.5	0.7265	116.8	23.6	0.7150	121.8	23.6	0.7323	125.5	24.3	0.7411
R2	153.3	29.9	0.8335	141.8	27.4	0.7988	137.8	27.9	0.7715	147.2	28.5	0.8056	151.4	29.4	0.8130
R3	163.5	31.9	0.8553	151.5	29.2	0.8228	145.9	29.4	0.7899	157.2	30.4	0.8298	161.6	31.3	0.8367
P2	77.1	13.6	0.8706	71.6	12.7	0.8262	53.8	9.4	0.6787	71.0	12.9	0.7996	73.5	13.5	0.8153
P3	48.3	7.0	0.8556	44.6	6.6	0.8051	48.6	8.3	0.8558	44.1	6.9	0.7753	45.8	7.4	0.7931
P4	34.0	3.6	0.8380	31.1	3.4	0.7803	34.2	4.8	0.8387	30.7	3.8	0.7470	32.0	4.3	0.7672

Table A3 Kinetic parameter of PPC-PCHC thermal decomposition by Coats-Redfern method

Method No.	β=2 K min^{-1}			β=2 K min^{-1}			β=2 K min^{-1}			β=2 K min^{-1}			β=2 K min^{-1}		
	E /kJ mol^{-1}	ln A	R^2	E /kJ mol^{-1}	ln A		E /kJ mol^{-1}	ln A	R^2	E /kJ mol^{-1}	ln A		E /kJ mol^{-1}	ln A	R^2
F1	103.6	19.9	0.7771	121.1	23.7	0.8923	123.4	24.5	0.8807	127.1	25.4	0.8515	112.0	21.7	0.9123
F2	158.6	33.0	0.8998	179.6	37.2	0.9620	183.8	38.4	0.9602	191.6	40.0	0.9546	166.3	33.9	0.9795
F3	267.0	58.1	0.9774	253.6	54.0	0.9723	260.3	55.7	0.9774	273.3	58.3	0.9885	234.8	49.0	0.9861
D1	187.0	34.9	0.8289	232.8	46.6	0.8316	225.6	45.8	0.8825	222.1	45.5	0.9053	215.5	45.8	0.8289
D2	237.2	48.6	0.8568	243.3	49.0	0.9249	247.5	50.1	0.9052	256.5	56.7	0.8619	205.7	40.0	0.8821
D3	264.8	53.6	0.8884	270.0	53.6	0.9456	275.3	54.9	0.9298	286.7	57.2	0.8959	229.5	43.8	0.9117
D4	246.3	49.2	0.8682	252.1	49.5	0.9326	256.7	50.7	0.9142	266.5	52.7	0.8742	213.5	40.3	0.8929
A2	0.0	5.7	0.8968	67.7	12.0	0.9520	69.3	12.7	0.9377	72.8	13.7	0.9086	56.6	9.9	0.9176
A3	41.6	5.7	0.8824	42.1	6.1	0.9447	43.1	6.7	0.9286	45.4	7.5	0.8962	34.5	4.9	0.9020
R1	103.2	19.5	0.8157	106.4	20.1	0.8974	108.2	20.8	0.8730	111.7	21.7	0.8191	88.7	16.3	0.8409
R2	121.1	23.1	0.8655	123.8	23.5	0.9319	126.2	24.3	0.9131	131.2	25.5	0.8726	104.1	19.1	0.8894
R3	127.9	24.3	0.8809	130.4	24.6	0.9417	133.0	25.5	0.9249	138.7	26.8	0.8891	109.9	20.1	0.9040
P2	47.1	6.7	0.7850	29.3	2.9	0.8541	29.9	3.4	0.8221	51.1	8.5	0.7900	39.5	5.8	0.8055
P3	28.4	2.2	0.7471	44.6	6.6	0.8051	49.4	6.8	0.7795	30.9	3.9	0.7544	23.1	2.0	0.7586
P4	19.0	-0.2	0.7001	19.7	0.5	0.8226	20.1	1.0	0.7859	20.8	1.4	0.7104	14.9	-0.1	0.6959

Table A4 Kinetic parameter of PCHC-PPC-PCHC thermal decomposition by Coats-Redfern method

Method No.	β=2 K min^{-1}			β=2 K min^{-1}			β=2 K min^{-1}			β=2 K min^{-1}			β=2 K min^{-1}		
	E /kJ mol^{-1}	ln A	R^2	E /kJ mol^{-1}	ln A		E /kJ mol^{-1}	ln A	R^2	E /kJ mol^{-1}	ln A		E /kJ mol^{-1}	ln A	R^2
F1	166.8	33.7	0.8598	172.7	34.9	0.8768	179.2	36.2	0.8729	162.8	32.6	0.9082	119.8	22.8	0.8696
F2	223.6	46.8	0.9198	231.1	48.1	0.9343	240.6	49.8	0.9377	217.1	44.6	0.9573	162.1	32.1	0.9406
F3	269.5	57.5	0.9465	282.4	59.7	0.9596	295.1	61.9	0.9668	277.6	57.9	0.9808	216.3	43.9	0.9807
D1	205.4	41.0	0.7062	217.2	43.2	0.7304	225.2	44.6	0.7233	219.4	43.3	0.7938	168.2	31.6	0.7665
D2	227.6	45.4	0.7413	240.4	47.8	0.7649	249.6	49.4	0.7597	242.2	47.6	0.8257	186.5	34.9	0.8038
D3	256.2	50.5	0.7831	270.3	53.0	0.8058	280.9	54.8	0.8025	271.2	52.5	0.8622	209.7	38.5	0.8454
D4	291.6	58.3	0.8232	302.1	59.9	0.8403	312.5	61.5	0.8320	286.1	55.6	0.8745	211.1	38.7	0.8276
A2	0.0	0.4	0.7857	68.6	12.0	0.8114	71.5	12.9	0.8107	68.4	12.3	0.8708	51.2	8.3	0.8465
A3	40.1	5.1	0.7582	42.5	6.1	0.7873	44.5	6.8	0.7872	42.4	6.5	0.8524	30.8	3.8	0.8165
R1	98.1	17.8	0.6856	103.9	19.3	0.7116	107.8	20.2	0.7046	104.9	19.7	0.7778	79.1	13.8	0.7428
R2	116.4	21.5	0.7489	123.1	23.1	0.7736	127.9	24.1	0.7696	123.6	23.2	0.8349	94.2	16.5	0.8105
R3	123.5	22.7	0.7696	130.4	24.3	0.7938	135.6	25.4	0.7907	130.8	24.4	0.8529	99.9	17.3	0.8315
P2	44.4	5.8	0.6386	47.2	6.9	0.6685	49.1	7.6	0.6620	47.6	7.4	0.7407	34.6	4.4	0.6853
P3	26.5	1.6	0.5828	28.3	2.6	0.6172	29.5	3.1	0.6117	28.5	3.1	0.6952	19.8	1.0	0.6107
P4	17.6	-0.7	0.5171	18.9	0.2	0.5562	19.7	0.8	0.5523	19.0	0.8	0.6391	12.3	-0.9	0.5145
